# Genetic Risk, BMI Status, BMI Change Patterns, and the Risk of Steatotic Liver Disease and Liver Enzyme Elevation in Chinese Adults

**DOI:** 10.3390/nu16234212

**Published:** 2024-12-06

**Authors:** Juan Yang, Chan Tian, Maojie Liu, Haiyan Guo, Fei Lin, Yang Ding, Wentao Yao, Jiahao Zhang, Jingyi Fan, Chengxiao Yu, Jing Lu, Qun Zhang

**Affiliations:** 1 Department of Epidemiology, Center for Global Health, School of Public Health, Nanjing Medical University, Nanjing 211166, China; juanyang0223@163.com (J.Y.); tc2419626380@163.com (C.T.); liumaojie99@163.com (M.L.); guohaiyan0227@163.com (H.G.); linfei930@163.com (F.L.); dingy1595@163.com (Y.D.); wentao.yao@foxmail.com (W.Y.); zhangjh0507@126.com (J.Z.); 2 Health Management Center, Gusu School, The Affiliated Suzhou Hospital of Nanjing Medical University, Suzhou Municipal Hospital, Suzhou 234099, China; fanjingyi@njmu.edu.cn; 3 Health Management Center, The First Affiliated Hospital of Nanjing Medical University, Nanjing 210029, China; yuchengxiao@njmu.edu.cn

**Keywords:** steatotic liver disease, liver enzyme elevation, genetic susceptibility, BMI change pattern

## Abstract

**Background/Objectives:** Whether an increased genetic risk of steatotic liver disease (SLD) can be offset by maintaining a healthy weight remains unknown. We aimed to clarify the associations among the body mass index (BMI) and its change patterns with SLD and assess whether genetic susceptibility can modify these associations in Chinese people. **Methods:** A total of 10,091 and 6124 participants from the Health Omics Preventive Examination (HOPE) Program were enrolled in cross-sectional and follow-up analyses, respectively. BMI change patterns were defined according to the BMI at baseline and the last follow-up visit. Genetic risk was estimated using the polygenic risk score (PRS) derived from variants in *PNPLA3*, *TM6SF2*, *MBOAT7*, and *GCKR*. Data were analyzed using logistic regression models and Cox proportional-hazards models. **Results:** The analyses of the BMI and genetic risk simultaneously showed a dose–response association with the risk of SLD (*p*-trend < 0.001). Significant interactions between BMI and PRS were found for alanine aminotransferase (ALT) elevation (*p* = 0.007) and aspartate aminotransferase (AST) elevation (*p* < 0.001). Weight loss led to a 71%, 60%, and 67% lower risk of SLD, ALT elevation, and AST elevation, compared with stable overweight/obesity. A significant interaction between the genetic risk and BMI change patterns in ALT elevation was observed (*p* = 0.008). The absolute risk reductions associated with weight loss were greater for participants at a high genetic risk (26.60, 12.29, and 9.31 per 100 person years for SLD, ALT elevation, and AST elevation, respectively). **Conclusions:** Maintaining a healthy weight reduces the liver injury risk among all individuals, and the risk reduction is greater among the subset with a high genetic risk of SLD.

## 1. Introduction

Steatotic liver disease (SLD) is the most prevalent chronic liver disease worldwide, affecting approximately 30% of the population [1]. The prevalence of SLD in the Chinese population is approximately 30% and it has shown an escalating trend over the years [2]. Obesity is an established risk factor for SLD, and more than 50% of obese adults will progress to SLD [3]. Previous studies have shown that trajectories of weight gain during young adulthood are associated with a greater SLD risk in midlife, and weight loss has been shown to not only reduce hepatic steatosis but also to improve biomarkers of hepatic steatosis [4,5,6]. However, these studies mainly focused on the impact of weight changes through early and middle adulthood on the SLD risk in American and European populations, and few studies have investigated the association among short-term weight changes and SLD in Asian individuals. A previous Japanese study showed that short-term weight gain was related to an increased risk of SLD [7], but only non-obese people were studied. Therefore, establishing a comprehensive framework to analyze the impact of the BMI and its change patterns on the risk of SLD and liver enzyme elevation will provide a theoretical foundation for the development of targeted prevention and intervention strategies for Chinese people.

While environmental risk factors, such as the BMI, are the primary contributors to the risk of SLD [8], genetic factors have been estimated to account for 20–37% of the variance in the presence of SLD [9]. In the past decade, genome-wide association studies (GWASs) have identified several variants associated with hepatic steatosis [10]. Although an individual single-nucleotide polymorphism only partially explains the genetic variation in SLD, a polygenic risk score (PRS), derived from well-established hereditary variations in *PNPLA3*, *TM6SF2*, *MBOAT7*, and *GCKR*, has been shown to be effective in estimating an individual’s genetic predisposition to SLD [11]. Moreover, prior studies found that the PRS for hepatic steatosis was statistically significantly related to higher plasma ALT and AST levels among Caucasian children [12,13]. However, the degree to which a genetic predisposition to hepatic steatosis contributes to SLD and liver enzyme elevation in Chinese adults remains unclear. Although the interactions between genes and adiposity on SLD and ALT levels have been previously explored [14], whether and the extent to which weight loss can offset the increased genetic risk of SLD and liver enzyme elevation is not yet clear among Chinese individuals.

To help to fill these gaps, we conducted a cross-sectional and follow-up study to explore the associations among the BMI, and its short-term change, and the genomic information related to hepatic steatosis and the risk of SLD and aminotransferase elevation among 12,007 participants from the Health Omics Preventive Examination (HOPE) Program. The HOPE study was a multi-center, prospective cohort study conducted in China. The current study enrolled the health examination population who visited the health management center of the First Affiliated Hospital of Nanjing Medical University in Jiangsu. The main objectives of the study were to (1) characterize the independent impacts of the BMI status, BMI change patterns, and genetic factors on liver injuries; (2) test the joint associations of these factors and gene–environment interactions in the risk of SLD and liver enzyme elevation; and (3) assess the degree to which weight loss or a stable normal weight might reduce the risk of SLD and liver enzyme elevation.

## 2. Materials and Methods

### 2.1. Study Design and Population

All 12,007 individuals aged 18 and older were invited to participate in the HOPE study from 2019 to 2023, with annual follow-up. The study excluded participants with missing data on genetic factors (N = 888), BMI (N = 328), liver enzymes (N = 75), SLD (N = 378), covariates (N = 92), and excessive alcohol consumption or other known liver etiologies (N = 155). The baseline cross-sectional evaluation therefore included 10,091 eligible participants. After further excluding participants without follow-up BMI or outcome information, as well as those with SLD or ALT or AST elevations at baseline, 6124 participants remained for the SLD analysis, 6550 participants were used for the ALT elevation analysis, and 6989 were used for the AST elevation analysis in the follow-up study. Supplemental Figure S1 displays the inclusion and exclusion process regarding the study population. This study was conducted in compliance with the Declaration of Helsinki and was approved by the ethics committee of Nanjing Medical University. All subjects signed a written informed consent form prior to their involvement in the study.

### 2.2. Genotyping and PRS Calculation

Genomic DNA was extracted from venous blood from each participant. After extraction, the genomic DNA was immediately stored at −80 °C. The genotyping of *PNPLA3* rs738409, *TM6SF2* rs58542926, *MBOAT7* rs641738, and *GCKR* rs1260326 was carried out. To examine the overall genetic burden on the SLD risk, the PRS was calculated by summing the number of effect alleles at each locus, each multiplied by its corresponding weight coefficient as reported in published studies [11], and detailed information is provided in Supplemental Table S1. The genetic risk of SLD was categorized as low, intermediate, or high based on the tertiles of the PRS.

### 2.3. Measurement of Exposure: BMI and BMI Change Pattern

All participants underwent a standardized clinical evaluation at an annual medical visit, including detailed anthropometric measurements, performed by a trained clinical investigator. The body mass index was computed as the weight in kilograms divided by the height in meters squared. Underweight (BMI < 18.5) was rare; thus, it was not considered as a separate category in the main analyses. Therefore, the BMI was classified into 3 categories based on the Chinese guidelines [15]: less than 24 (normal weight), 24 to less than 28 (overweight), and 28 or greater (obese).

BMI change patterns were established based on the BMI at baseline and the last follow-up visit: “stable normal weight” (BMI_baseline_ < 24 kg/m^2^ and BMI_last visit_ < 24 kg/m^2^), “weight loss” (BMI_baseline_ ≥ 24 kg/m^2^ and BMI_last visit_ < 24 kg/m^2^), “weight gain” (BMI_baseline_ < 24 kg/m^2^ and BMI_last visit_ ≥ 24 kg/m^2^), and “stable overweight/obese” (BMI_baseline_ ≥ 24 kg/m^2^ and BMI_last visit_ ≥ 24 kg/m^2^).

### 2.4. Measurement of Hepatic Steatosis and Liver Enzymes

In accordance with guidelines and existing studies, SLD was diagnosed when hepatic steatosis was present [16]. In this study, ultrasonography and computed tomography-guided liver examinations were conducted in all participants by trained doctors. A liver-to-spleen ratio (L/S) < 1.0 was used for the diagnosis of hepatic steatosis in the baseline cross-sectional study [17], and US was used as the diagnostic method in the follow-up study. Venous blood was tested by laboratory biochemistry for liver enzyme values. A liver enzyme elevation was defined by adopting sex-specific cut-off values. ALT elevation was defined as having an ALT value of greater than 33 UI/L in males and 25 UI/L in females [18], and AST elevation was defined as having an AST value of greater than 30 UI/L in males and 26 UI/L in females [19].

### 2.5. Statistical Analyses

In the cross-sectional analysis, logistic regression models were used to estimate the odds ratios (ORs) and 95% confidence intervals (CIs) between the BMI categories, genetic risk, and SLD, as well as the liver enzyme elevation risk. The potential non-linear associations between the SLD risk, genetic risk score, and BMI were evaluated using restricted cubic splines with three knots. In addition, the joint effects of the BMI categories and genetic risk on liver injuries were examined. The interaction was evaluated by including multiplicative interaction terms in the logistic regression models.

In the prospective analysis, a Cox proportional-hazards model was used to estimate the association between BMI change patterns and liver injuries. To explore the combined associations of the BMI change patterns and genetic risk for SLD, eight combined groups were created by crossing four BMI change patterns with two genetic risk categories, using those with a high genetic risk and stable overweight/obesity as the reference group. This study further assessed the interactions between the BMI change patterns and genetic risk of SLD in liver injuries. The absolute risk reduction in SLD and aminotransferase elevation incidence in these given groups were computed to evaluate the benefits of weight loss or a stable normal weight over a 3-year period. To derive the 95% confidence intervals for the absolute risk reduction, 1000 bootstrap samples were generated from the estimation dataset. Sensitivity analyses were conducted to evaluate the stability of the results by (1) reclassifying the genetic risk groups according to the quartiles or quintiles of the PRS and (2) removing individuals with pre-existing diabetes, hypertension, or dyslipidemia to prevent any potential reverse association.

In the aforementioned analyses, all models were adjusted for age, sex, smoking status, drinking status, education level, regular physical activity, and chronic diseases (hypertension, diabetes, hyperlipidemia). A two-sided *p* value of less than 0.05 was regarded as indicative of a significant difference. R (version 4.3.1) was used to perform all statistical analyses.

## 3. Results

### 3.1. Characteristics of Study Participants

The baseline characteristics of the participants are listed in Table 1. Overall, the average age (SD) of all 10,091 participants was 38.29 (10.39) year, and almost half were males (48.79%). During a median follow-up of 2.92 years (interquartile range: 2.25–3.83 years), 1318 out of the 6124 subjects developed SLD, 860 out of the 6550 subjects developed an ALT elevation, and 702 out of the 6989 subjects developed an AST elevation. Supplemental Table S2 shows the baseline characteristics of the subjects participating in the follow-up study according to their BMI change patterns. In general, 4072 (66.49%) participants remained at a normal weight (classified as “stable normal weight” group), 611 (9.98%) became overweight/obese (classified as “weight gain” group), 160 (2.61%) transitioned from being overweight/obese to a normal weight (classified as “weight loss” group), and 1281 (20.92%) remained overweight/obese (classified as “overweight/obesity” group).

### 3.2. Genetic Associations with SLD and Liver Enzyme Elevation Risk

**Figure 1:**
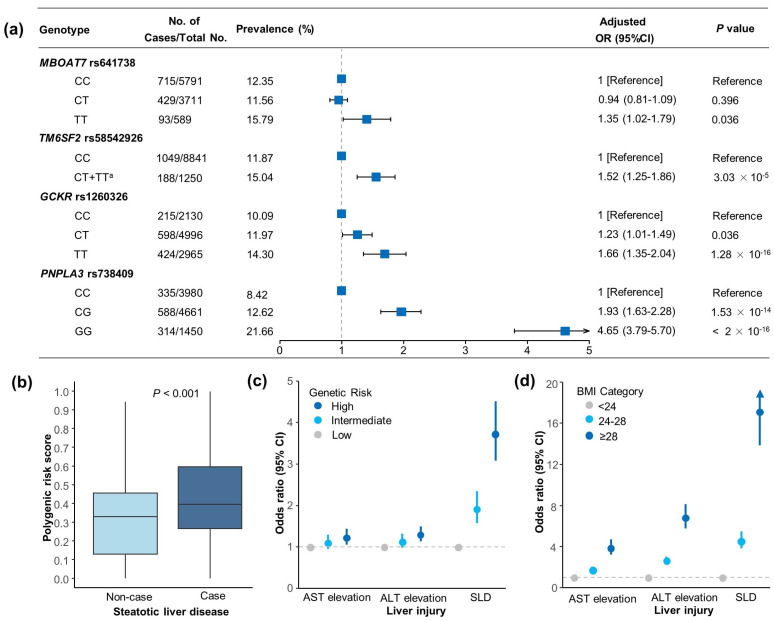
Effects of genetic factor and BMI category on the risk of steatotic liver disease and liver enzyme elevation in the HOPE cohort. (**a**) Associations between individual SNPs and SLD. (**b**) Polygenic risk scores among SLD cases versus non-cases. In the boxplot, the median is represented by horizontal lines, the interquartile range is indicated by the upper and lower edges of the box, and the whiskers display the maximum and minimum values for each group. The *p* value was derived from the Mann–Whitney U test, and *p* < 0.001 indicates that the difference in the polygenic risk score distribution between the case group and the non-case group was statistically significant. (**c**) Associations of genetic risk categories with liver injuries. (**d**) Associations of BMI categories with liver injuries. Genetic risk groups were established based on the tertiles of the PRS (low, intermediate, and high). ORs and 95% CIs were determined after controlling for age, sex, smoking status, drinking status, education level, regular physical activity, hypertension, diabetes, hyperlipidemia, BMI (SNP and genetic risk analysis), or genetic risk (BMI category analysis). ^a^ Because only 39 participants had the *TM6SF2* rs58542926 TT genotype, the CT and TT genotypes were combined into one explanatory variable, “CT+TT”. OR, odds ratio; 95% CI, 95% confidence interval; SLD, steatotic liver disease; BMI, body mass index; ALT, alanine aminotransferase; AST, aspartate aminotransferase; SNPs, single-nucleotide polymorphisms.

The associations of *PNPLA3*, *TM6SF2*, *MBOAT7*, and *GCKR* variants and the SLD risk were tested and the four single-nucleotide polymorphisms (SNPs) were all associated with the SLD risk. The strongest evidence of an association was found between rs738409 in the *PNPLA3* variant and SLD, with ORs of 1.93 (1.63–2.28) for CG and 4.65 (3.79–5.70) for the GG genotype (Figure 1a). The result showed that the risk of SLD increased linearly with the increase in the PRS; see Supplemental Figure S2 (*p* for overall <0.001, *p* for non-linearity = 0.142). Participants with SLD exhibited a higher PRS compared to those without SLD (*p* < 0.001) (Figure 1b). After additional adjustment for the BMI, participants in the high-genetic-risk group (top tertile) had a 273% higher risk of SLD (OR: 3.73, 95% CI 3.08–4.51), a 30% higher risk of ALT elevation (OR: 1.30, 95% CI: 1.13–1.49), and a 23% higher risk of AST elevation (OR: 1.23, 95% CI: 1.05–1.43) compared to those in the low-genetic-risk group (bottom tertile) (Figure 1c, Supplemental Table S3).

### 3.3. Associations of BMI with SLD and Liver Enzyme Elevation Risk

In the analysis of the dose–response relationship, the BMI showed a non-linear association with the SLD risk, following an approximately J-shaped curve (*p* for non-linear <0.001), with a significantly increasing trend in the OR for each 1 kg/m^2^ increase in the BMI when the BMI was greater than 23 kg/m^2^ (Supplemental Figure S3). The multivariable-adjusted ORs (95 CI%) for the SLD risk were 4.57 (3.81–5.48) for overweight and 17.13 (13.84–21.19) for obese when compared with a normal weight (Figure 1d, Supplemental Table S4).

### 3.4. Joint Association and Interaction of BMI and PRS in SLD and Liver Enzyme Elevation Risk

**Figure 2:**
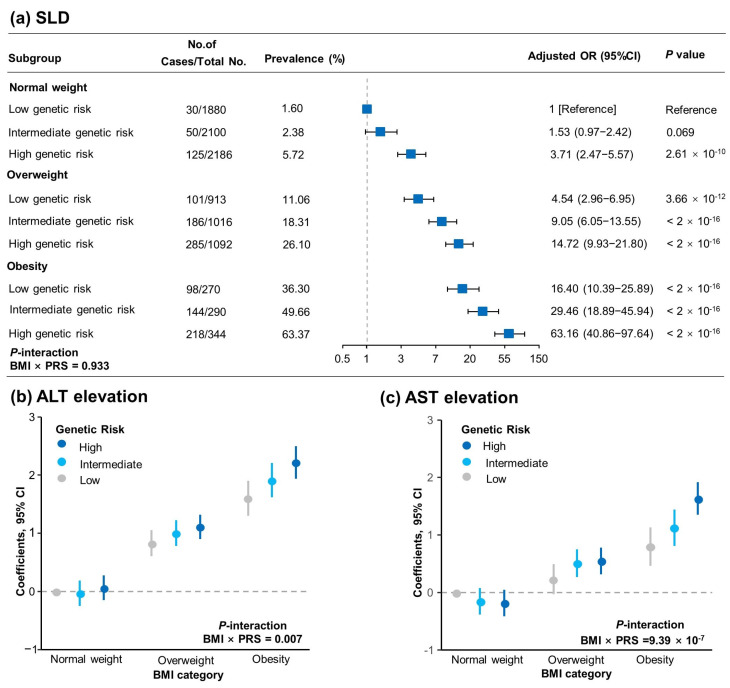
Joint associations of genetic risk, BMI category, and risk of SLD (**a**), ALT elevation (**b**), and AST elevation (**c**). Subjects with low genetic risk and normal weight were used as the reference group. ORs were calculated by logistic regression, adjusted for age, sex, smoking status, drinking status, education level, regular physical activity, hypertension, diabetes, and hyperlipidemia.

As the PRS and BMI groups were combined, the SLD and liver enzyme elevation risk grew as both factors rose (Figure 2). Among all of the categories analyzed, individuals with both a high genetic risk and obesity exhibited the highest liver injury risk. Compared with those with a low genetic susceptibility to SLD and normal weight, having a high genetic risk and obesity was linked to an increased risk of SLD (OR: 63.16, 95% CI: 40.86–97.64), ALT elevation (OR: 9.21, 95% CI: 6.96–12.18), and AST elevation (OR: 5.10, 95% CI: 3.86–6.82). Moreover, the risk of SLD and aminotransferase elevation remained consistently lower in individuals with a normal weight than in those with obesity, within each category of genetic risk. Significant interactions between the genetic risk and BMI categories regarding aminotransferase elevation were observed (*p* for interaction = 0.007 for ALT elevation; *p* for interaction = 9.39×10^−7^ for AST elevation), indicating that the risk-increasing effect of the genetic risk on aminotransferase elevation was amplified by increasing adiposity. However, no significant interaction was found between the PRS and BMI categories for SLD (*p* for interaction = 0.933).

### 3.5. Relations of BMI Change Pattern and SLD and Aminotransferase Elevation

The results showed that individuals who lost weight and transitioned from overweight/obesity to a normal weight had a 71% lower risk of SLD (HR, 0.29, 95% CI, 0.20–0.44), a 60% lower risk of ALT elevation (HR, 0.40, 95% CI, 0.25–0.66), and a 67% lower risk of AST elevation (HR, 0.33, 95% CI, 0.18–0.60) compared with stable overweight/obese individuals. Moreover, participants who gained weight, moving from a normal weight to overweight/obesity, had a 114% higher risk of SLD (HR 2.14, 95% CI, 1.81–2.53) and a 62% higher risk of ALT elevation (HR 1.62, 95% CI, 1.30–2.01) compared with those who maintained a normal weight (Table 2).

### 3.6. Joint Effect and Interaction of BMI Change Pattern and PRS in Incident SLD and Aminotransferase Elevation Risk

**Figure 3:**
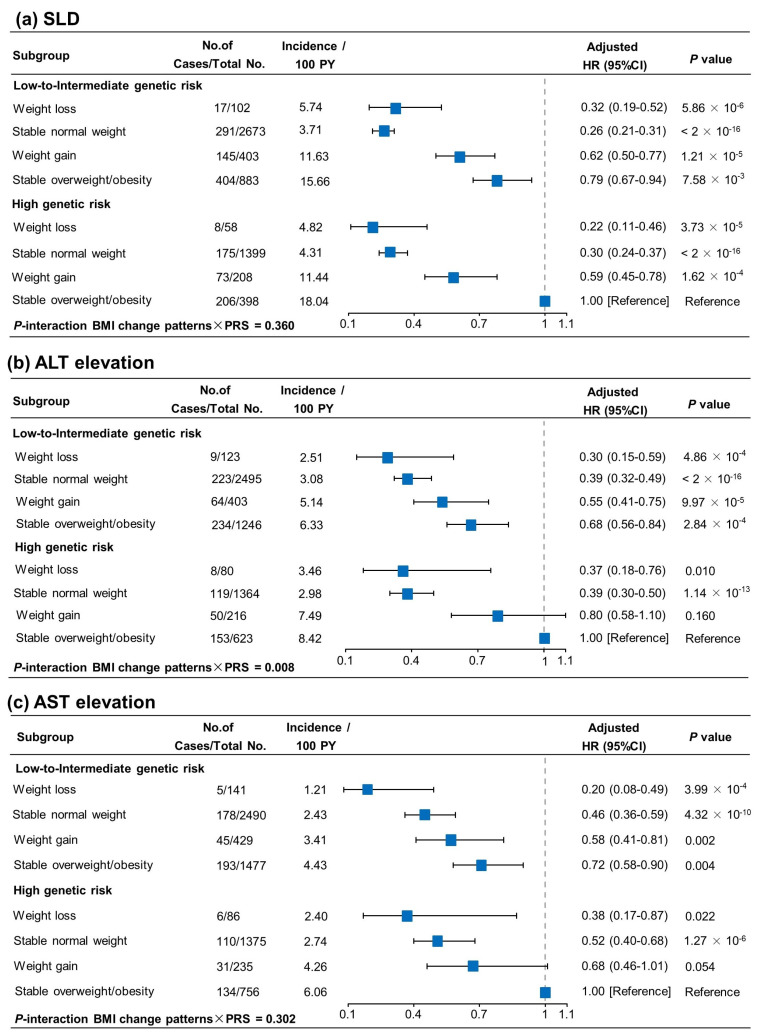
Joint associations of genetic risk, BMI change pattern, and risk of SLD (**a**), ALT elevation (**b**), and AST elevation (**c**). Individuals having a high genetic risk and stable overweight/obesity served as the reference group. Risk estimates were adjusted for age, sex, smoking status, drinking status, education level, regular physical activity, hypertension, diabetes, and hyperlipidemia. PY, person years.

The tendencies of the risk of SLD and aminotransferase elevation among participants with different BMI change patterns and genetic risks of SLD are presented in Figure 3. As compared with those with stable overweight/obesity and a high genetic risk, weight loss was associated with a 78% (HR 0.22, 95% CI, 0.11–0.46) lower risk of SLD among those at a high genetic risk, and a stable normal weight was associated with a 70% (HR 0.30, 95% CI, 0.24–0.37) lower risk of SLD among those at a high genetic risk. Likewise, individuals experienced a gradually decreased liver enzyme elevation risk across the four BMI change patterns in both the low-to-intermediate- and high-genetic-risk categories. An interaction between the BMI change patterns and PRS was observed only for ALT elevation (*p* for interaction = 0.008).

### 3.7. Standardized Three-Year Absolute Risk of SLD and Aminotransferase Elevation According to Genetic Risk and BMI Change Patterns

**Figure 4:**
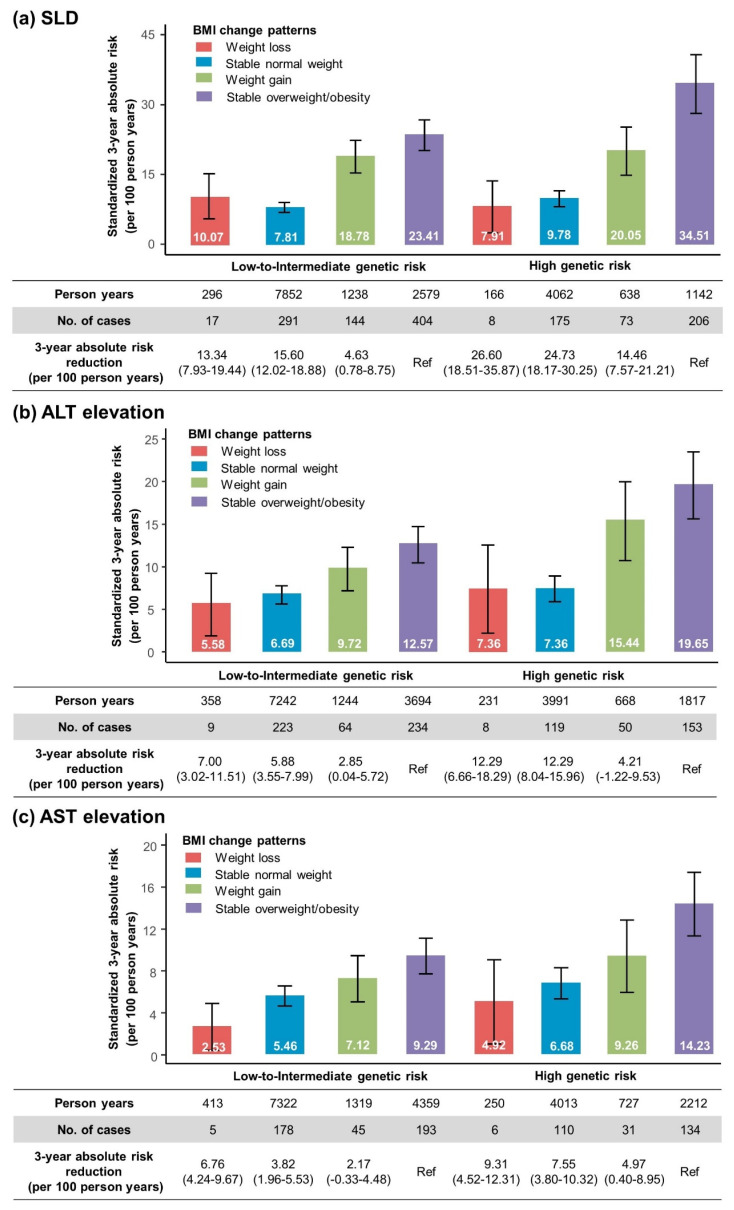
Standardized 3-year absolute risk (per 100 person years) for SLD (**a**), ALT elevation (**b**), and AST elevation (**c**) according to BMI change patterns in different genetic risk groups. The 3-year absolute risks were age- and sex-adjusted. Genetic risk was classified into low-to-intermediate (tertiles 1–2) and high (the top tertile) based on the tertiles of the polygenic risk score. Error bars represent 95%CI of 3-year absolute risk. The HRs were obtained using Cox proportional-hazards regression with adjustment for age, sex, smoking status, drinking status, education level, regular physical activity, hypertension, diabetes, and hyperlipidemia.

The age- and sex- adjusted 3-year absolute risk of SLD was lower for those with a low-to-intermediate genetic risk (spanning from 7.81 to 23.41 per 100 person years) than for those with a high genetic risk (spanning from 7.91 to 34.51 per 100 person years), but was higher for those with stable overweight/obesity (spanning from 23.41 to 34.51 per 100 person years) than for those with weight loss or a stable normal weight (ranging from 7.81 to 10.07 per 100 person years) at both low-to-intermediate and high genetic risk levels. When those with stable overweight/obesity were used as the reference group, the adjusted 3-year absolute SLD risk reductions associated with weight loss were 13.34 (95% CI, 7.93–19.44) per 100 person years for the low-to-intermediate genetic risk category and 26.60 (95% CI, 18.51–35.87) per 100 person years for the high genetic risk category. Moreover, when people maintained a normal weight, the 3-year absolute SLD risk reductions in the low-to-intermediate and high genetic risk categories were 15.60 (95% CI, 12.02–18.88) and 24.73 (95% CI, 18.17–30.25) per 100 person years, respectively. A comparable pattern of results was observed for the adjusted 3-year absolute risk estimates of ALT and AST elevation. Among individuals who experienced weight loss or maintained a normal weight, the 3-year absolute risk reduction was larger in the high genetic risk category than the low-to-intermediate genetic risk category (Figure 4). The results were successfully replicated in the sensitivity analyses, and similar 3-year absolute risk trends of incident SLD and aminotransferase elevation according to the genetic risk and BMI change patterns were observed (Supplemental Tables S5–S10).

## 4. Discussion

In this study conducted on a population of Chinese adults, the research revealed that (1) obesity and weight gain were positively correlated with a higher risk of SLD and aminotransferase elevation, whereas weight loss was negatively associated; (2) the greater absolute risk reductions for liver injuries associated with maintaining a normal weight or weight loss occurred among those with a high genetic risk of SLD. The current study offers compelling evidence supporting the benefits of maintaining a normal weight for the prevention of SLD and aminotransferase elevation in the whole population, particularly among individuals having a high genetic susceptibility to SLD.

It has been well recognized that the BMI status and absolute weight changes are associated with the risk of SLD. Previous studies showed that absolute weight gain was related to SLD and weight loss was associated with the remission of hepatic steatosis in both obese and non-obese patients [6,20,21]. However, few studies have reported the associations of BMI changes in various patterns defined by the cutoff values of the China BMI classification and liver injury. The current study demonstrated that recent short-term BMI change patterns were closely related to the development of SLD and liver enzyme elevation. Specifically, weight gain from a normal weight to overweight/obesity was a significant risk factor for liver injury, whereas weight loss from overweight/obesity to a normal weight substantially reduced the risk of liver injury. The findings indicate that health examination populations could be categorized based on their BMI change patterns to prevent SLD in healthcare services and in clinical practice.

Previous studies that included subjects of European ancestry found that the PRS-HFC could accurately identify patients with SLD and refined the diagnostic performance for liver damage [11,22,23]. Among East Asian populations, Thomas et al. found that the PRS-HFC derived from Europeans was also useful in assessing the HCC risk [24]. Taken together, the previous findings support that the PRS-HFC, as a robust genetic instrument, is predictive of SLD. In this study with Chinese individuals as the subjects, the PRS-HFC was also independently and robustly associated with a higher risk of SLD, with a high genetic risk score having a 3.73-fold higher risk of SLD compared with a low genetic risk score. Furthermore, our results showed that individuals at a high genetic risk of SLD had a higher risk of ALT and AST elevation than those at a low-to-intermediate genetic risk. This was consistent with a prior study conducted in Denmark that reported that the PRS for hepatic steatosis was robustly associated with higher plasma concentrations of ALT and AST [12].

There is existing evidence that interactions between lifestyle factors (e.g., obesity and alcohol use) and a genetic predisposition to liver damage are present [14,25,26]. In this study, BMI–PRS interactions were present for liver enzyme elevation, and the BMI change pattern–PRS interaction was statistically significant only for ALT elevation. The results suggest that adiposity played a pivotal role in shaping the impact of the genetic risk of SLD on liver enzyme elevation, which was in line with a previous study that found that the association between the PRS and increased ALT was amplified as the BMI increased [27]. In contrast with some observations revealing the interaction between the genetic risk and BMI in SLD [28], no significant interaction for SLD was found. This discrepancy is likely because of the ethnic differences in the allele frequencies and possibly inadequate power to detect it. The biological mechanisms behind the observed interaction results are not clear. Future studies are warranted to further explore the mechanisms.

The results regarding the joint effects of the BMI and PRS demonstrate that having a normal weight could reduce the liver injury risk at all levels of genetic susceptibility to SLD. Among participants with high genetic susceptibility to SLD, this study even found no significant risk effect of the genetic factor on liver enzyme elevation when the subject had a normal weight. Likewise, in the follow-up analysis, individuals who had a high genetic risk of SLD and lost weight or maintained a normal weight had a lower risk of developing SLD and aminotransferase elevation compared with those who had a low-to-intermediate genetic risk but gained weight or maintained overweight/obesity. These findings indicate the potential benefits of a stable normal weight regardless of the genetic risk. Furthermore, it was observed that, compared with individuals at a low-to-intermediate genetic risk, the beneficial effects of weight loss and a stable normal weight were more evident in subjects at a high genetic risk, who experienced greater 3-year absolute risk reductions regarding liver injuries. The results indicate that maintaining a normal weight could partly offset the deleterious effect of the genetic susceptibility to SLD, which once again underscores the importance of maintaining a normal weight in preventing SLD and aminotransferase elevation, particularly for individuals with a high genetic risk of SLD.

The strength of the current study is that it offers a thorough understanding of the associations of the BMI, short-term BMI change patterns, and genetic risk with liver injuries in Chinese populations, with a cross-sectional and prospective design. This study also has some limitations. First, although the BMI is most widely used and easily obtained in clinical practice, the fat distribution may be more informative [29]. A second limitation is that ultrasound is not the “gold standard” for the identification of hepatic steatosis; however, it is neither feasible nor ethical to undertake liver biopsies in large cohorts of healthy people. Third, the small proportion of the sample that experienced weight loss limited the accuracy of the calculations. Fourth, the beta coefficients associated with the genetic risk used to construct the PRS-HFC were derived from European population data, which may have resulted in racial differences. However, relevant data in Asian populations are lacking. Fifth, the study only included Chinese populations; therefore, our findings cannot be generalized to other populations. Finally, as more genetic variants are identified [10], the variance explained by genetics and PRS estimates will be improved.

## 5. Conclusions

In Chinese individuals, maintaining a healthy weight could significantly lower the SLD and aminotransferase elevation risk, regardless of the genetic susceptibility to SLD. Weight control could provide a greater benefit for individuals with a high genetic risk of SLD compared to those with a low genetic risk.

## Figures and Tables

**Table 1:** Baseline characteristics of the study population.

Characteristic	Total (N = 10,091)	Non-Steatotic Liver Disease (N = 8854)	Steatotic Liver Disease (N = 1237)	*p* Value †
Sex, N(%)				
Male	4923 (48.79)	3952 (44.64)	971 (78.50)	<0.001
Female	5168 (51.21)	4902 (55.36)	266 (21.50)	
Age (years), Mean ± SD	38.29 ± 10.39	38.22 ± 10.40	38.81 ± 10.30	0.058
Education level, N(%)				
High school or below	609 (6.04)	529 (5.97)	80 (6.47)	0.537
College degree or above	9482 (93.96)	8325 (94.03)	1157 (93.53)	
Smoking status, N(%)				
Never	8457 (83.81)	7565 (85.44)	892 (72.11)	<0.001
Ever	1634 (16.19)	1289 (14.56)	345 (27.89)	
Drinking status, N(%)				
Never	8460 (83.84)	7537 (85.13)	923 (74.62)	<0.001
Ever	1631 (16.16)	1317 (14.87)	314 (25.38)	
Regular physical activity, N(%)				
No	6808 (67.47)	5886 (66.48)	922 (74.54)	<0.001
Yes	3283 (32.53)	2968 (33.52)	315 (25.46)	
Body mass index (kg/m^2^), Mean ± SD	23.38 ± 3.34	22.85 ± 2.95	27.22 ± 3.45	<0.001
BMI category, N(%)				
<24	6166 (61.10)	5961 (67.33)	205 (16.57)	<0.001
24–28	3021 (29.94)	2449 (27.66)	572 (46.24)	
≥28	904 (8.96)	444 (5.01)	460 (37.19)	
ALT (IU/L), Median (IQR)	16.80 (11.80–25.90)	15.50 (11.30–22.60)	38.80 (26.40–57.50)	<0.001
AST (IU/L), Median (IQR)	19.70 (16.80–23.90)	19.20 (16.50–22.80)	27.00 (21.70–35.00)	<0.001
Hypertension, N(%)				
No	8602 (85.24)	7787 (87.95)	815 (65.89)	<0.001
Yes	1489 (14.76)	1067 (12.05)	422 (34.11)	
Diabetes, N(%)				
No	9833 (97.44)	8706 (98.33)	1127 (91.11)	<0.001
Yes	258 (2.56)	148 (1.67)	110 (8.89)	
Hyperlipidemia, N(%)				
No	7779 (77.09)	7204 (81.36)	575 (46.48)	<0.001
Yes	2312 (22.91)	1650 (18.64)	662 (53.52)	
Genetic risk category, N (%)				
Low	3063 (30.35)	2834 (32.01)	229 (18.51)	<0.001
Intermediate	3406 (33.75)	3026 (34.18)	380 (30.72)	
High	3622 (35.89)	2994 (33.82)	628 (50.77)	

† *p* values were calculated using independent-sample *t* test or Mann–Whitney U test for continuous variables and χ^2^ test for categorical variables. BMI, body mass index; ALT, alanine aminotransferase; AST, aspartate aminotransferase.

**Table 2:** The association between the BMI change pattern and the risk of liver injury.

Outcome	Risk Reduction: Weight Loss Versus Stable Overweight/Obesity †			Risk Raise: Weight Gain Versus Stable Normal Weight ‡		
	Total No. (Cases)	HR (95%CI) §	*p* Value	Total No. (Cases)	HR (95%CI) §	*p* Value
Steatotic liver disease	1441 (635)	0.29 (0.20–0.44)	3.37 × 10^−9^	4683 (683)	2.14 (1.81–2.53)	<2 × 10^−16^
ALT elevation	2072 (404)	0.40 (0.25–0.66)	2.94 × 10^−4^	4478 (456)	1.62 (1.30–2.01)	1.77 × 10^−5^
AST elevation	2460 (338)	0.33 (0.18–0.60)	2.77 × 10^−4^	4529 (364)	1.27 (0.98–1.64)	0.072

† Stable overweight or obese group was designated as the reference group. ‡ Stable normal weight group was designated as the reference group. § HRs and 95% CIs were adjusted for age, sex, smoking status, drinking status, education level, regular physical activity, hypertension, diabetes, hyperlipidemia, and genetic risk category. BMI, body mass index; ALT, alanine aminotransferase; AST, aspartate aminotransferase; HR, hazard ratio; 95%CI, 95% confidence interval.

## Data Availability

The datasets of the study are available from the authors upon valid request. The data are not accessible to the public because of laboratory policies.

## Supplementary Materials

The following supporting information can be downloaded at: https://www.mdpi.com/article/10.3390/nu16234212/s1, Figure S1. Flowchart of the study; Figure S2. Linear relationships between polygenic risk score and steatotic liver disease risk.; Figure S3. Linear relationships between BMI and steatotic liver disease risk.; Table S1. Information on four SNPs used to build polygenic risk score; Table S2. Participant characteristics by BMI change pattern among 6124 participants without steatotic liver disease at baseline; Table S3. Association of genetic risk category with SLD, ALT elevation, and AST elevation; Table S4. Association of BMI categories with SLD, ALT elevation, and AST elevation; Table S5. Risk of incident SLD according to BMI change pattern within each genetic risk category based on quartiles or quintiles of polygenic risk score (PRS); Table S6. Risk of incident ALT elevation according to BMI change pattern within each genetic risk category based on quartiles or quintiles of polygenic risk score (PRS); Table S7. Risk of incident AST elevation according to BMI change pattern within each genetic risk category based on quartiles or quintiles of polygenic risk score (PRS); Table S8. Risk of incident SLD according to BMI change pattern within each genetic risk category with exclusion of chronic diseases at baseline; Table S9. Risk of incident ALT elevation according to BMI change pattern within each genetic risk category with exclusion of chronic diseases at baseline; Table S10. Risk of incident AST elevation according to BMI change pattern within each genetic risk category with exclusion of chronic diseases at baseline.
