# Supplementary material for: Genetic Risk, BMI Status, BMI Change Patterns, and the Risk of Steatotic Liver Disease and Liver Enzyme Elevation in Chinese Adults
Source: Nutrients. 2024 Dec 6;16(23):4212. doi: 10.3390/nu16234212 (PMC11644279; doi:10.3390/nu16234212)
Supplement: Supplementary file 1 [file nutrients-16-04212-s001.zip › nutrients-3355633-supplementary.pdf]

Supplementary material

Supplementary Figure S1. Flowchart of the study.

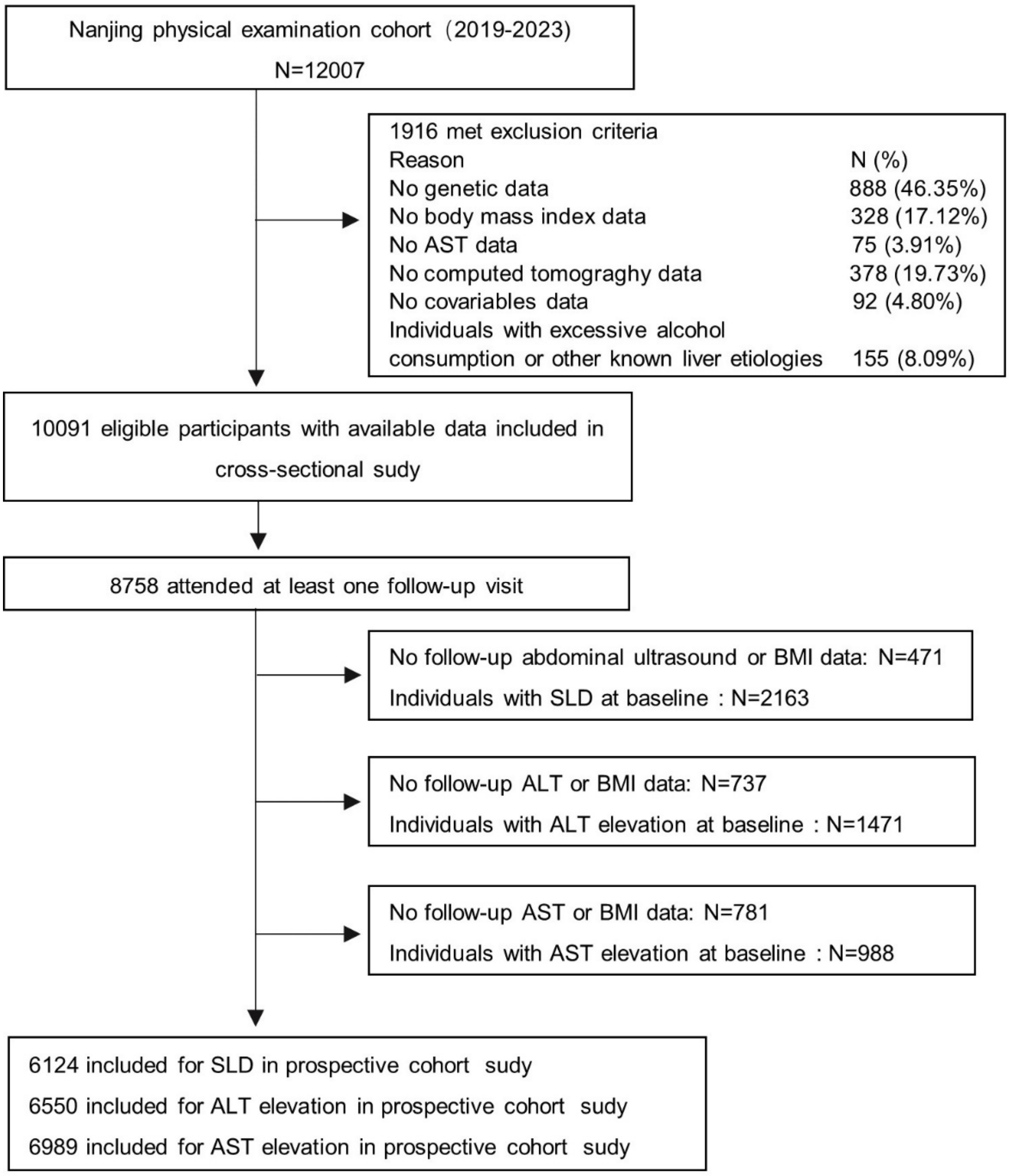

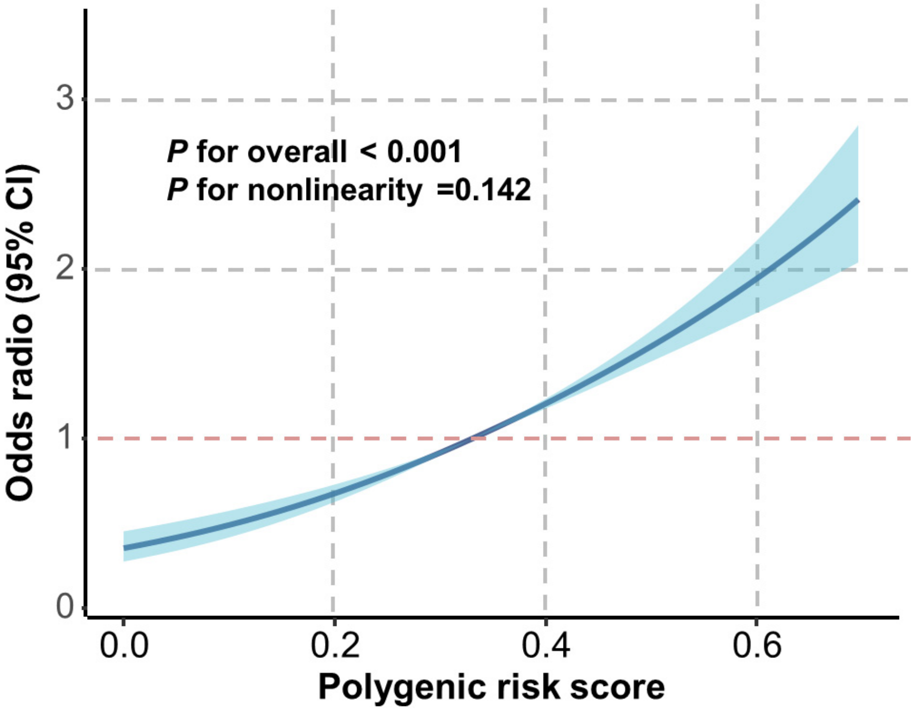

Supplementary Figure S2. Linear relationships between polygenic risk score and steatotic liver disease risk. Risk estimates were adjusted for age, sex, smoking status, drinking status, education level, regular physical activity, hypertension, diabetes, hyperlipidemia and BMI. 95% CI, 95% confidence interval.

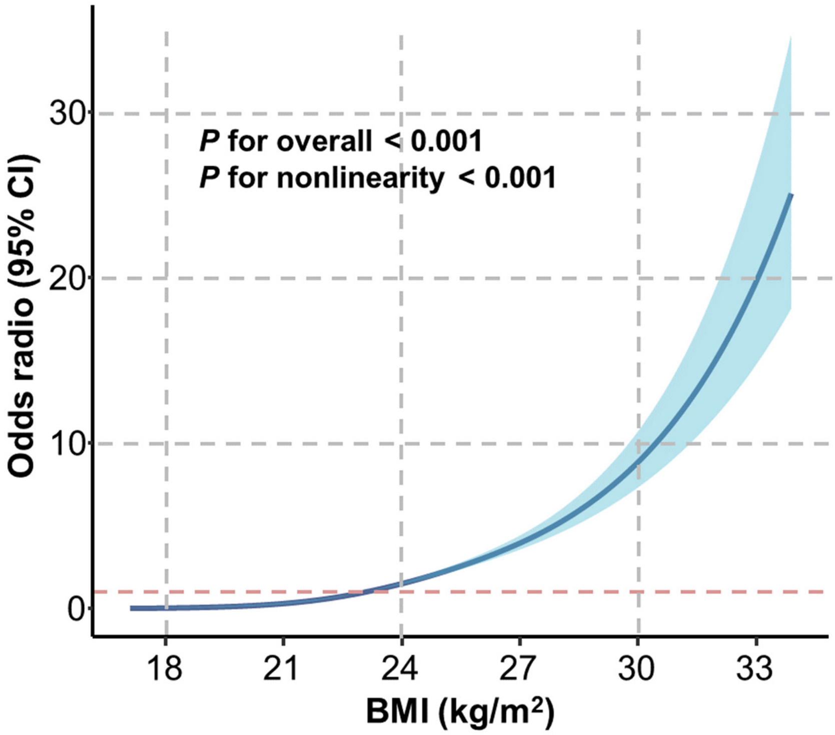

Supplementary Figure S3. Linear relationships between BMI and steatotic liver disease risk. Risk estimates were adjusted for age, sex, smoking status, drinking status, education level, regular physical activity, hypertension, diabetes, hyperlipidemia and polygenic risk score. BMI, body mass index; 95% CI, 95% confidence interval.

Supplementary Table S1. Information on four SNPs used to build polygenic risk score.

SNP ID	Mapped gene	Location	Reference allele	Effect allele	Beta	Effect allele frequency
rs1260326	GCKR	2:27508073	C	T	0.0647	0.5414
rs641738	MBOAT7	19:54173068	C	T	0.0629	0.2422
rs58542926	TM6SF2	19:19268740	C	T	0.2738	0.0639
rs738409	PNPLA3	22:43928847	C	G	0.2657	0.3746

Supplementary Table S2. Participant characteristics by BMI change patterns among 6124 people without SLD at baseline. †

Characteristic	Weight loss (N=160, 2.61%)	Stable normal weight (N=4072, 66.49%)	Weight gain (N=611, 9.98%)	Stable overweight/obesity (N=1281, 20.92%)	*P* value ‡
**Sex, N(%)**					
Male	77 (48.13)	1123 (27.58)	303 (49.59)	892 (69.63)	<0.001
Female	83 (51.88)	2949 (72.42)	308 (50.41)	389 (30.37)	
**Age(years), Mean±SD**	38.96 ± 11.37	37.02 ± 9.49	35.54 ± 8.56	38.62 ± 10.23	<0.001
**Education level, N(%)**					
High school or below	12 (7.50)	110 (2.70)	16 (2.62)	70 (5.46)	<0.001
College degree or above	148 (92.50)	3962 (97.30)	595 (97.38)	1211 (94.54)	
**Smoking status, N(%)**					
Never	138 (86.25)	3786 (92.98)	538 (88.05)	981 (76.58)	<0.001
Ever	22 (13.75)	286 (7.02)	73 (11.95	300 (23.42)	
**Drinking status, N(%)**					
Never	149 (93.13)	3697 (90.79)	532 (87.07)	950 (74.16)	<0.001
Ever	11 (6.88)	375 (9.21)	79 (12.93)	331 (25.84)	
**Regular physical activity, N(%)**					
No	115 (71.88)	2841 (69.77)	398 (65.14)	755 (58.94)	<0.001
Yes	45 (28.13)	1231 (30.23)	213 (34.86)	526 (41.06)	
**Hypertension, N(%)**					
No	133 (83.13)	3826 (93.96)	573 (93.78)	1058 (82.59)	<0.001
Yes	27 (16.88)	246 (6.04)	38 (6.22)	223 (17.41)	
**Diabetes, N(%)**					
No	152 (95.00)	4040 (99.21)	607 (99.35)	1266 (98.83)	<0.001
Yes	8 (5.00)	32 (0.79)	4 (0.65)	15 (1.17)	
**Hyperlipidemia, N(%)**					

No	134 (83.75)	3612 (88.70)	527 (86.25)	991 (77.36)	<0.001
Yes	26 (16.25)	460 (11.30)	84 (13.75)	290 (22.64)	
***PNPLA3* rs738409, N(%)**					
CC	67 (41.88)	1600 (39.29)	254 (41.57)	574 (44.81)	0.017
CG	75 (46.88)	1899 (46.64)	277 (45.34)	563 (43.95)	
GG	18 (11.25)	573 (14.07)	80 (13.09)	144 (11.24)	
***TM6SF2* rs58542926, N(%)**					
CC	139 (86.88)	3607 (88.58)	536 (87.73)	1128 (88.06)	0.829
CT+TT	21 (13.13)	465 (11.42)	75 (12.27)	153 (11.94)	
***MBOAT7* rs641738, N(%)**					
CC	96 (60.00)	2369 (58.18)	340 (55.65)	716 (55.89)	0.411
CT	56 (35.00)	1482 (36.39)	230 (37.64)	478 (37.31)	
TT	8 (5.00)	221 (5.43)	41 (6.71)	87 (6.79)	
***GCKR* rs1260326, N(%)**					
CC	36 (22.50)	857 (21.05)	125 (20.46)	298 (23.26)	0.353
CT	72 (45.00)	2033 (49.93)	315 (51.55)	642 (50.12)	
TT	52 (32.50)	1182 (29.03)	171 (27.99)	341 (26.62)	
**Genetic risk category, N (%)** §					
Low-to-Intermediate	102 (63.75)	2673 (65.64)	403 (65.96)	883 (68.93)	0.154
High	58 (36.25)	1399 (34.36)	208 (34.04)	398 (31.07)	
**Steatotic liver disease, N(%)**					
No	135 (84.38)	3606 (88.56)	394 (64.48)	671 (52.38)	<0.001
Yes	25 (15.63)	466 (11.44)	217 (35.52)	610 (47.62)	

† Percentages may not sum to 100 because of rounding.

‡ *P* values were calculated using one-way ANOVA test for equal variances, Kruskal-Wallis's test for unequal variances and $\chi2$ test for categorical variables.

§ Genetic risk was categorized into low-to- intermediate (tertiles 1-2) and high (the top tertile) according to distributions of polygenic risk score.

Supplementary Table S3. Association of genetic risk category with SLD, ALT elevation and AST elevation

Outcome	Genetic risk categories †	No. of Cases/Total	Prevalence (%)	Model 1		Model 2		Model 3	
				OR (95% CI)	*P* value	OR (95% CI)	*P* value	OR (95% CI)	*P* value
**SLD**									
	Low	229/3063	7.48	1.00 [Reference]	Reference	1.00 [Reference]	Reference	1.00 [Reference]	Reference
	Intermediate	380/3406	11.16	1.55 (1.30-1.85)	9.73E-07	1.72 (1.43-2.07)	8.74E-09	1.92 (1.57-2.34)	2.82E-10
	High	628/3622	17.34	2.69 (2.29-3.17)	< 2E-16	3.05 (2.56-3.63)	< 2E-16	3.73 (3.08-4.51)	< 2E-16
	P for trend				< 2E-16		< 2E-16		< 2E-16
**ALT elevation**									
	Low	515/3063	16.81	1.00 [Reference]	Reference	1.00 [Reference]	Reference	1.00 [Reference]	Reference
	Intermediate	612/3406	17.97	1.07 (0.94-1.22)	0.307	1.11 (0.97-1.27)	0.135	1.13 (0.98-1.31)	0.082
	High	731/3622	20.18	1.26 (1.11-1.43)	3.55E-04	1.27 (1.12-1.45)	3.34E-04	1.30 (1.13-1.49)	1.63E-04
	P for trend						2.93E-04		1.49E-04
**AST elevation**									
	Low	350/3063	11.43	1.00 [Reference]	Reference	1.00 [Reference]	Reference	1.00 [Reference]	Reference
	Intermediate	415/3406	12.18	1.07 (0.92-1.20)	0.413	1.09 (0.93-1.27)	0.276	1.10 (0.94-1.29)	0.216
	High	494/3622	13.64	1.22 (1.05-1.41)	0.009	1.21 (1.04-1.40)	0.013	1.23 (1.05-1.43)	0.009
	P for trend				8.00E-03		0.012		0.008

Model 1: adjusted for age and sex;

Model 2: model 1+ smoking status, drinking status, education level, regular physical activity, hypertension, diabetes, hyperlipidemia;

Model 3: model 2+ BMI.

† Defined as: low (lowest tertile), medium (second tertile) and high (highest tertile).

Definition of abbreviations: SLD, steatotic liver disease; ALT, alanine aminotransferase; AST, aspartate aminotransferase; BMI, body mass index; OR, odds ratio; 95%CI, 95% confidence interval.

Supplementary Table S4. Association of BMI categories with SLD, ALT elevation and AST elevation

Outcome	BMI categories	No. of Cases/Total	Prevalence (%)	Model 1		Model 2		Model 3	
				OR (95% CI)	*P* value	OR (95% CI)	*P* value	OR (95% CI)	*P* value
**SLD**									
	<24	205/6166	3.32	1.00 [Reference]	Reference	1.00 [Reference]	Reference	1.00 [Reference]	Reference
	24 to <28	572/3021	18.93	5.13 (4.31-6.12)	< 2E-16	4.43 (3.70-5.30)	< 2E-16	4.57 (3.81-5.48)	< 2E-16
	≥28	460/904	50.88	22.65 (18.59-27.60)	< 2E-16	15.79 (12.81-19.46)	< 2E-16	17.13 (13.84-21.19)	< 2E-16
	P for trend				< 2E-16		< 2E-16		< 2E-16
**ALT elevation**									
	<24	580/6166	9.41	1.00 [Reference]	Reference	1.00 [Reference]	Reference	1.00 [Reference]	Reference
	24 to <28	801/3021	26.51	2.93 (2.58-3.32)	< 2E-16	2.65 (2.33-3.02)	< 2E-16	2.66 (2.33-3.02)	< 2E-16
	≥28	477/904	52.77	8.87 (7.54-10.45)	< 2E-16	6.84 (5.75-8.13)	< 2E-16	6.84 (5.75-8.13)	< 2E-16
	P for trend				< 2E-16		< 2E-16		< 2E-16
**AST elevation**									
	<24	487/6166	7.9	1.00 [Reference]	Reference	1.00 [Reference]	Reference	1.00 [Reference]	Reference
	24 to <28	477/3021	15.79	1.93 (1.67-2.23)	< 2E-16	1.75 (1.51-2.03)	1.19E-13	1.75 (1.51-2.03)	1.13E-13
	≥28	295/904	32.63	5.03 (4.22-6.00)	< 2E-16	3.88 (3.21-4.68)	< 2E-16	3.88 (3.21-4.68)	< 2E-16
	P for trend				< 2E-16		< 2E-16		< 2E-16

Model 1: adjusted for age and sex;

Model 2: model 1+ smoking status, drinking status, education level, regular physical activity, hypertension, diabetes, hyperlipidemia;

Model 3: model 2+ genetic risk categories.

Definition of abbreviations: BMI, body mass index; SLD, steatotic liver disease; ALT, alanine aminotransferase; AST, aspartate aminotransferase; OR, odds ratio; 95%CI, 95% confidence interval.

Supplementary Table S5. Risk of incident SLD according to BMI change pattern within each genetic risk category based on quartiles or quintiles of polygenic risk score (PRS).

Subgroup	Low-to-Intermediate genetic risk				High genetic risk			
	Weight loss	Stable normal weight	Weight gain	Stable overweight or obese	Weight loss	Stable normal weight	Weight gain	Stable overweight/obesity
**Based on quartiles of PRS †**								
No. of Cases / Total No.	17/119	328/3024	167/459	464/1003	8/41	138/1048	50/152	146/278
Incidence / 100 person-year	5.00	3.70	11.79	15.9	6.56	4.54	10.87	18.2
HR (95% CI)	0.27 (0.16-0.45)	0.25 (0.20-0.30)	0.59 (0.47-0.74)	0.79 (0.65-0.95)	0.28 (0.14-0.57)	0.31 (0.24-0.39)	0.58 (0.42-0.81)	1.00 [Reference]
P Value	3.46E-07	<2E-16	6.56E-06	0.012	4.66E-04	<2E-16	0.001	Reference
Adjusted 3-years absolute risk (95% CI)	9.36 (4.85-13.87)	7.96 (6.95-8.98)	19.25 (15.93-22.58)	25.11 (21.82-28.39)	9.12 (2.59-15.64)	9.93 (7.93-11.94)	19.08 (13.18-24.99)	32.88 (25.73-40.02)
Adjusted 3-years absolute risk reduction (95% CI)	15.75 (10.21-21.76)	17.14 (13.54-20.53)	5.85 (1.91-9.83)	Ref	23.76 (14.47-34.62)	22.94 (15.08-29.20)	13.79 (6.25-21.59)	Ref
**Based on quintiles of PRS ‡**								
No. of Cases / Total No.	18/130	350/3238	178/497	493/1064	7/30	116/834	39/114	117/217

Incidence / 100 person-year	4.81	3.68	11.69	15.95	8.05	4.82	11.05	18.6
HR (95% CI)	0.25 (0.15-0.41)	0.24 (0.20-0.30)	0.60 (0.47-0.76)	0.80 (0.65-0.97)	0.35 (0.16-0.75)	0.34 (0.26-0.44)	0.56 (0.39-0.81)	1.00 [Reference]
P Value	5.61E-08	<2E-16	2.13E-05	0.027	0.007	6.04E-16	0.002	Reference
Adjusted 3-years absolute risk (95% CI)	8.72 (4.64-12.81)	7.94 (6.96-8.91)	19.41 (16.17-22.65)	25.66 (22.43-28.88)	11.2 (2.56-19.83)	10.62 (8.26-12.97)	18.49 (11.98-25.01)	32.43 (24.38-40.49)
Adjusted 3-years absolute risk reduction (95% CI)	16.93 (12.12-22.20)	17.72 (14.59-20.98)	6.25 (2.31-9.91)	Ref	21.24 (11.26-35.17)	21.82 (13.32-29.28)	13.94 (4.70-22.27)	Ref

HRs and 95% CIs were estimated with adjustment for age, sex, education level, smoking status, drinking status, regular physical activity, hypertension, diabetes and hyperlipidemia.

† Defined by quartiles of PRS: low (the lowest quartile), intermediate (quartile 2-3) and high (the highest quartile).

‡ Defined by quintiles of PRS: low (the bottom quintile), intermediate (quintiles 2-4) and high (the top quintile).

Definition of abbreviations: BMI, body mass index; SLD, steatotic liver disease; HR, hazard ratio; 95%CI, 95% confidence interval.

Supplementary Table S6. Risk of incident ALT elevation according to BMI change pattern within each genetic risk category based on quartiles or quintiles of polygenic risk score (PRS).

Subgroup	Low-to-Intermediate genetic risk				High genetic risk			
	Weight loss	Stable normal weight	Weight gain	Stable overweight or obese	Weight loss	Stable normal weight	Weight gain	Stable overweight/obesity
**Based on quartiles of PRS †**								
No. of Cases / Total No.	10/145	246/2837	83/462	277/1426	7/58	96/1022	31/157	110/443
Incidence / 100 person-year	2.41	2.95	5.79	6.57	4.02	3.22	6.49	8.51
HR (95% CI)	0.28 (0.15-0.53)	0.37 (0.29-0.47)	0.60 (0.45-0.80)	0.69 (0.55-0.86)	0.41 (0.19-0.88)	0.41 (0.31-0.54)	0.67 (0.44-0.99)	1.00 [Reference]
P Value	1.10E-04	2.57E-16	5.97E-04	0.001	0.022	5.19E-10	0.048	Reference
Adjusted 3-years absolute risk (95% CI)	5.49 (2.06-8.92)	6.45 (5.48-7.42)	10.93 (8.39-13.47)	13.35 (11.28-15.42)	7.87 (1.88-13.86)	8.29 (6.37-10.21)	13.83 (8.62-19.05)	19.78 (15.11-24.45)
Adjusted 3-years absolute risk reduction (95% CI)	7.86 (4.24-12.30)	6.90 (4.75-9.07)	2.42 (-0.41-5.16)	Ref	11.91 (5.23-19.07)	11.49 (6.52-15.96)	5.95 (-0.44-12.76)	Ref
**Based on quintiles of PRS ‡**								
No. of Cases / Total No.	10/161	264/3078	91/504	303/1541	7/42	78/781	23/115	84/328
Incidence / 100 person-year	2.15	2.91	5.85	6.65	5.65	3.46	6.44	8.8
HR (95% CI)	0.23 (0.12-0.45)	0.34 (0.26-0.44)	0.58 (0.43-0.79)	0.66 (0.52-0.84)	0.52 (0.24-1.13)	0.42 (0.31-0.58)	0.58 (0.37-0.93)	1.00 [Reference]
P Value	1.40E-05	2.82E-16	4.19E-04	7.49E-04	0.098	9.34E-08	0.023	Reference

Adjusted 3-years absolute risk (95% CI)	4.79 (1.8-7.78)	6.31 (5.39-7.22)	11.05 (8.6-13.51)	13.48 (11.49-15.48)	11.59 (2.7-20.47)	9.7 (7.23-12.17)	14.4 (8.14-20.67)	20.97 (15.34-26.6)
Adjusted 3-years absolute risk reduction (95% CI)	8.69 (5.39-12.67)	7.18 (5.04-9.10)	2.43 (-0.20-5.10)	Ref	9.38 (1.13-18.48)	11.27 (8.04-15.96)	6.57 (-2.20-13.96)	Ref

HRs and 95% CIs were estimated with adjustment for age, sex, education level, smoking status, drinking status, regular physical activity, hypertension, diabetes and hyperlipidemia.

† Defined by quartiles of PRS: low (the lowest quartile), intermediate (quartile 2-3) and high (the highest quartile).

‡ Defined by quintiles of PRS: low (the bottom quintile), intermediate (quintiles 2-4) and high (the top quintile).

Definition of abbreviations: BMI, body mass index; ALT, alanine aminotransferase; HR, hazard ratio; 95%CI, 95% confidence interval.

Supplementary Table S7. Risk of incident AST elevation according to BMI change pattern within each genetic risk category based on quartiles or quintiles of polygenic risk score (PRS).

Subgroup	Low-to-Intermediate genetic risk				High genetic risk			
	Weight loss	Stable normal weight	Weight gain	Stable overweight or obese	Weight loss	Stable normal weight	Weight gain	Stable overweight/obesity
**Based on quartiles of PRS †**								
No. of Cases / Total No.	7/167	198/2831	53/491	230/1699	4/60	90/1034	23/173	97/534
Incidence / 100 person-year	1.45	2.38	3.49	4.59	2.23	2.99	4.35	6.23
HR (95% CI)	0.23 (0.10-0.49)	0.42 (0.33-0.55)	0.56 (0.39-0.78)	0.70 (0.55-0.89)	0.33 (0.12-0.89)	0.54 (0.40-0.72)	0.64 (0.41-1.02)	1.00 [Reference]
P Value	1.49E-04	1.24E-10	7.42E-04	0.004	0.029	4.64E-05	0.059	Reference
Adjusted 3-years absolute risk (95% CI)	3.07 (0.78-5.37)	5.19 (4.32-6.07)	7.18 (5.15-9.22)	9.85 (8.2-11.5)	4.29 (0.01-8.57)	7.78 (5.91-9.66)	9.68 (5.51-13.86)	14.48 (10.87-18.1)
Adjusted 3-years absolute risk reduction (95% CI)	6.78 (4.07-9.89)	4.65 (2.78-6.33)	2.66 (0.36-5.13)	Ref	10.19 (5.22-16.44)	6.70 (2.84-10.25)	4.8 (-0.19-10.19)	Ref
**Based on quintiles of PRS ‡**								
No. of Cases / Total No.	7/182	214/3069	61/540	253/1832	4/45	74/796	15/124	74/401
Incidence / 100 person-year	1.32	2.37	3.67	4.68	3.03	3.23	3.89	6.35
HR (95% CI)	0.20 (0.09-0.43)	0.41 (0.31-0.54)	0.57 (0.40-0.80)	0.69 (0.53-0.89)	0.42 (0.15-1.16)	0.57 (0.41-0.80)	0.53 (0.30-0.92)	1.00 [Reference]

P Value								Reference
Adjusted 3-years absolute risk (95% CI)	2.78 (0.71-4.86)	5.23 (4.39-6.07)	7.63 (5.61-9.66)	10.17 (8.56-11.79)	5.97 (0.01-11.95)	8.63 (6.31-10.94)	8.71 (4.09-13.32)	14.42 (10.26-18.58)
Adjusted 3-years absolute risk reduction (95% CI)	7.39 (5.02-9.90)	4.94 (3.23-6.66)	2.54 (0.20-4.99)	Ref	8.45 (6.00-16.75)	5.79 (1.05-10.22)	5.71 (-0.001-11.84)	Ref

HRs and 95% CIs were estimated with adjustment for age, sex, education level, smoking status, drinking status, regular physical activity, hypertension, diabetes and hyperlipidemia.

† Defined by quartiles of PRS: low (the lowest quartile), intermediate (quartile 2-3) and high (the highest quartile).

‡ Defined by quintiles of PRS: low (the bottom quintile), intermediate (quintiles 2-4) and high (the top quintile).

Definition of abbreviations: BMI, body mass index; AST, aspartate aminotransferase; HR, hazard ratio; 95%CI, 95% confidence interval.

Supplementary Table S8. Risk of incident SLD according to BMI change pattern within each genetic risk category with exclusion of chronic diseases at baseline.

Subgroup	Low-to-Intermediate genetic risk †				High genetic risk †			
	Weight loss	Stable normal weight	Weight gain	Stable overweight or obese	Weight loss	Stable normal weight	Weight gain	Stable overweight/obesity
No. of Cases / Total No.	9/69	210/2247	112/324	241/579	5/41	120/1157	60/172	122/256
Incidence / 100 person-year	4.52	3.18	11.24	14.35	4.35	3.56	11.52	16.58
HR (95% CI)	0.27 (0.14-0.53)	0.23 (0.18-0.29)	0.60 (0.46-0.78)	0.74 (0.59-0.92)	0.23 (0.09-0.57)	0.26 (0.20-0.33)	0.66 (0.48-0.90)	1.00 [Reference]
P Value	1.43E-04	<2E-16	1.30E-04	0.008	0.001	<2E-16	0.008	Reference
Adjusted 3-years absolute risk (95% CI)	7.93 (2.68-13.18)	6.65 (5.57-7.73)	17.43 (13.65-21.21)	20.7 (16.98-24.42)	7.48 (0.81-14.15)	7.93 (6.22-9.64)	20.9 (14.85-26.96)	31.67 (24.35-38.99)
Adjusted 3-years absolute risk reduction (95% CI)	12.77 (6.74-10.55)	14.05 (9.55-17.65)	3.27 (-1.41-7.46)	Ref	24.19 (15.28-34.71)	23.74 (16.08-30.26)	10.77 (2.53-18.40)	Ref

HRs and 95% CIs were estimated with adjustment for age, sex, education level, smoking status, drinking status, regular physical activity.

† Defined by tertiles of PRS: low-to- intermediate (tertiles 1-2) and high (the top tertile).

Definition of abbreviations: BMI, body mass index; SLD, steatotic liver disease; HR, hazard ratio; 95%CI, 95% confidence interval.

Supplementary Table S9. Risk of incident ALT elevation according to BMI change pattern within each genetic risk category with exclusion of chronic diseases at baseline.

Subgroup	Low-to-Intermediate genetic risk †				High genetic risk †			
	Weight loss	Stable normal weight	Weight gain	Stable overweight or obese	Weight loss	Stable normal weight	Weight gain	Stable overweight/obesity
No. of Cases / Total No.	2/76	170/2078	50/316	114/673	3/49	90/1105	40/172	69/321
Incidence / 100 person-year	0.91	2.78	5.15	5.76	2.22	2.78	7.78	7.39
HR (95% CI)	0.12 (0.03-0.48)	0.41 (0.31-0.55)	0.63 (0.43-0.91)	0.73 (0.54-0.99)	0.30 (0.09-0.95)	0.41 (0.30-0.57)	1.00 (0.68-1.48)	1.00 [Reference]
P Value	0.003	1.61E-09	0.013	0.043	0.040	5.46E-08	0.996	Reference
Adjusted 3-years absolute risk (95% CI)	1.91 (-0.75-4.56)	6.40 (5.22-7.57)	10.00 (6.98-13.01)	11.33 (8.72-13.95)	5.10 (-0.73-10.92)	6.98 (5.29-8.67)	17.29 (11.37-23.22)	16.51 (11.78-21.25)
Adjusted 3-years absolute risk reduction (95% CI)	9.43 (5.93-13.11)	4.94 (2.29-7.38)	1.34 (-2.34-4.80)	Ref	11.42 (4.34-19.65)	9.54 (4.20-14.03)	-0.78 (-7.20-5.99)	Ref

HRs and 95% CIs were estimated with adjustment for age, sex, education level, smoking status, drinking status, regular physical activity.

† Defined by tertiles of PRS: low-to- intermediate (tertiles 1-2) and high (the top tertile).

Definition of abbreviations: BMI, body mass index; ALT, alanine aminotransferase; HR, hazard ratio; 95%CI, 95% confidence interval.

Supplementary Table S10. Risk of incident AST elevation according to BMI change pattern within each genetic risk category with exclusion of chronic diseases at baseline.

Subgroup	Low-to-Intermediate genetic risk †				High genetic risk †			
	Weight loss	Stable normal weight	Weight gain	Stable overweight or obese	Weight loss	Stable normal weight	Weight gain	Stable overweight/obesity
No. of Cases / Total No.	1/86	126/2073	33/335	77/769	1/55	81/1107	24/183	57/375
Incidence / 100 person-year	0.40	2.06	3.23	3.44	0.64	2.50	4.39	5.23
HR (95% CI)	0.08 (0.01-0.55)	0.43 (0.31-0.60)	0.59 (0.38-0.91)	0.62 (0.44-0.88)	0.12 (0.02-0.84)	0.52 (0.37-0.74)	0.84 (0.52-1.36)	1.00 [Reference]
P Value	0.010	5.01E-07	0.018	0.007	0.033	3.02E-04	0.482	Reference
Adjusted 3-years absolute risk (95% CI)	0.86 (-0.83-2.55)	4.84 (3.81-5.86)	6.94 (4.43-9.46)	7.32 (5.33-9.32)	1.34 (-1.3-3.97)	6.21 (4.58-7.83)	9.89 (5.63-14.15)	11.4 (7.76-15.04)
Adjusted 3-years absolute risk reduction (95% CI)	6.47 (3.95-9.06)	2.49 (0.37-4.25)	0.38 (-2.47-3.56)	Ref	10.06 (5.67-14.49)	5.20 (1.14-8.50)	1.52 (-3.84-7.19)	Ref

HRs and 95% CIs were estimated with adjustment for age, sex, education level, smoking status, drinking status, regular physical activity.

† Defined by tertiles of PRS: low-to- intermediate (tertiles 1-2) and high (the top tertile).

Definition of abbreviations: BMI, body mass index; AST, aspartate aminotransferase; HR, hazard ratio; 95%CI, 95% confidence interval.
